# Rutin Gel with Bone Graft Accelerates Bone Formation in a Rabbit Model by Inhibiting MMPs and Enhancing Collagen Activities

**DOI:** 10.3390/ph16050774

**Published:** 2023-05-22

**Authors:** Fahad F. Albaqami, Hassan N. Althurwi, Khalid M. Alharthy, Abubaker M. Hamad, Fatin A. Awartani

**Affiliations:** 1 Postgraduate Doctorate Program, Periodontics and Community Dentistry Department, College of Dentistry, King Saud University, Riyadh 11545, Saudi Arabia; 2 Pharmacology and Toxicology Department, College of Pharmacy, Prince Sattam bin Abdulaziz University, Al-Kharj 11942, Saudi Arabia; h.althurwi@psau.edu.sa (H.N.A.); k.alharthy@psau.edu.sa (K.M.A.); 3 Department of Nursing, College of Health Sciences and Nursing, Al-Rayan Colleges, Al-Madeena Al-Munowara 41411, Saudi Arabia; abkr.hamad@gmail.com; 4 Periodontics and Community Dentistry Department, College of Dentistry, King Saud University, P.O. Box 52500, Riyadh 11563, Saudi Arabia

**Keywords:** rutin, matrix metalloprotease (MMP), extracellular matrix (ECM), bone graft, collagen, natural product, flavonoid, in vivo, rabbit

## Abstract

Bone graft techniques are used to compensate for bone loss in areas with deficient regeneration. However, matrix metalloproteases (MMPs) can limit bone formation by degrading extracellular matrices, which are required for bone regrowth. Noteworthily, rutin is a natural flavonoid compound that inhibits the genetic expression of various MMPs. Therefore, rutin may serve as an inexpensive and stable alternative to the growth factors used to accelerate dental bone graft healing. This study aimed to evaluate the potential of mixing rutin gel with allograft bone to accelerate the healing of bone defects in an in vivo rabbit model. Bone defects were surgically induced in New Zealand rabbits (*n* = 3 per group) and subsequently treated with bone grafts along with rutin or control gel. Overall, treatment with rutin significantly prevented the expression of several MMPs and increased type III collagen in the gingiva around the surgical site. Additionally, rutin-treated animals showed enhanced bone formation with higher bone marrow content in the jawbone defect area compared with the control group. Taken together, these findings demonstrate that rutin gel, when added to bone grafts, quickly enhances bone formation and may serve as a suitable alternative to expensive growth factors for the same purpose.

## 1. Introduction

In periodontics, bone graft materials are used to encourage bone formation at defective sites, a process that increases the potential and predictability of bone regeneration. After the bone graft is placed at the planned site, bone formation is affected by the particle size of the bone graft, its physical and chemical properties, and the grafting procedure used [1]. Moreover, various types of growth factors can be added to the bone graft to trigger healing processes, resulting in accelerated bone formation and enhanced healing, which occurs through the induction of chemotaxis, as well as osteoblast proliferation and differentiation. Indeed, multiple studies showed that growth factors used in bone graft procedures have positive effects on bone formation [2,3,4].

Bone healing is a complex process in which diverse cell types, signaling pathways, and extracellular matrix (ECM) components interact [5]. In particular, matrix metalloproteases (MMPs) are a family of zinc-dependent endopeptidases that are responsible for the breakdown of the organic matrix [6]. MMPs control various biological activities, including cell migration, angiogenesis, wound healing, and tissue remodeling, by cleaving ECM proteins [7]; thus, they are associated with bone healing [8]. Moreover, extracellular matrices, such as type I collagen, are responsible for bone elasticity and toughness. In turn, type III collagen is a reconstituted collagen that plays a critical role in tissue damage repair, being highly expressed during injured tissue remodeling, promoting angiogenesis, and maintaining capillary function [9]. As vascularization can help accelerate the bone graft-mediated healing process [10], the MMP and ECM levels need to be reduced and enhanced, respectively, to support and increase the bone graft success rate.

**Figure 1:**
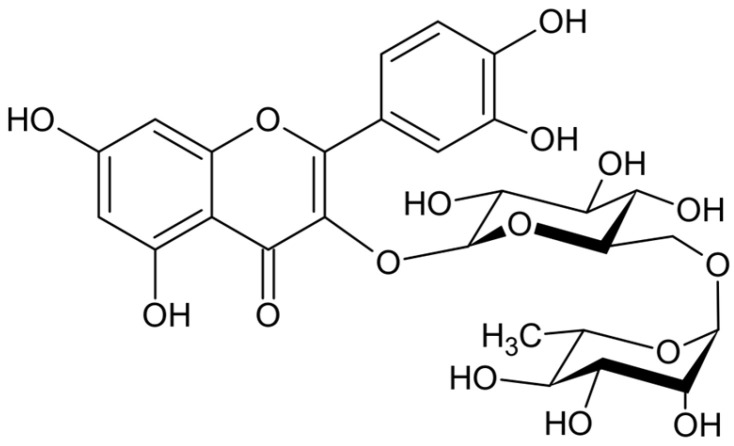
Chemical structure of rutin.

Various methods have been investigated for controlling MMP activity and expression, including the application of small-molecule inhibitors, gene therapy, and alteration of native MMP inhibitors [11]. Noteworthily, some bioactive molecules present in natural products can inhibit the MMP downstream pathways; thus, these molecules can represent valuable therapeutic tools for pathological conditions, including inflammatory processes and autoimmune diseases (such as osteoarthritis, periodontitis, and fibrotic disorders) [12]. Several studies have shown that rutin (Figure 1), a polyphenolic flavonoid found in fruits and vegetables, has several pharmacological benefits which are similar to other flavonoid compounds for treating different diseases, such as osteoporosis, cancer, diabetes, hypertension, viral infections, bacterial infections, and depression. Furthermore, rutin exerts powerful anti-inflammatory effects, decreases MMP expression, inhibits osteoclast formation, and promotes the proliferation and differentiation of osteoblasts. Unlike other flavonoids, rutin is non-toxic and non-oxidizable [13,14,15,16]. Hence, the safety, stability, and cost-effectiveness of rutin make it a suitable replacement for currently used growth factors.

Rutin is poorly soluble in aqueous solvents, which hinders its application in clinical settings due to reduced bioavailability. Therefore, we used the proniosomal gel to improve rutin solubility and bioavailability. In addition, using gel will aid in extending rutin’s pharmacological effects due to the controlled release properties of gel formulations. A further advantage of using proniosomal gel is that it can penetrate physiological barriers [17]. Thus, adding rutin gel to the defect area may inhibit the release of MMPs from the surrounding tissue into the defect area containing the bone graft.

The present study aims to provide an easy, simple, and rapid pathway to achieve desired results through bone grafting in vivo using rutin as a replacement for growth factors. Our study findings are expected to pave the way for the methodological development of practical dentistry, which will ultimately reduce the economic burden of the treatments and improve patient outcomes.

## 2. Results

The pilot study revealed that at the end of the sixth week, the bone healing process was still incomplete, whereas the healing process and new bone formation were almost completed 12 weeks after just tooth extraction (without intervention).

### 2.1. Gene Expression

Briefly, six New Zealand rabbits (*n* = 3) were randomly divided into two groups following unilateral lower first premolar tooth extraction: the control group received vehicle gel (without rutin) and the treated group received rutin gel, which was mixed with bone graft to fill the bone defect. As described in Section 4, the animals were euthanized after six weeks of treatment, and specimens were collected for analysis of gene expression and histomorphometry.

**Figure 2:**
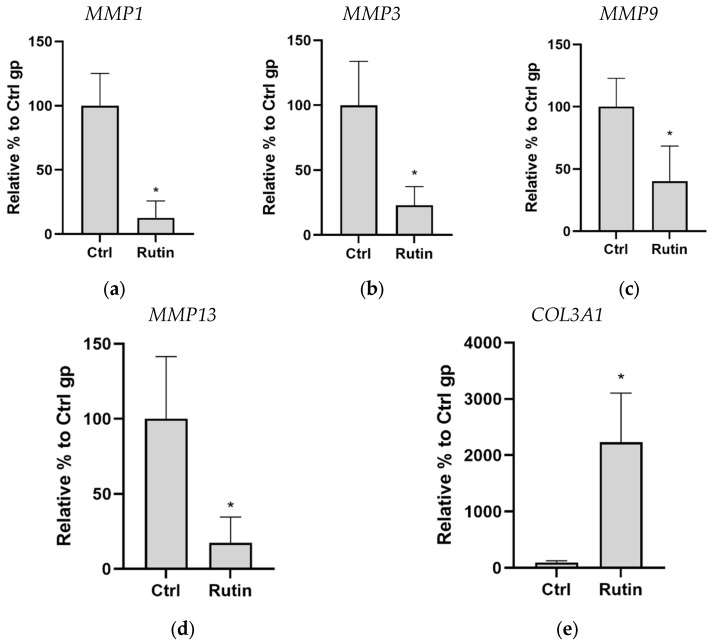
Effect of rutin gel treatment on *MMP* and *COL3A1* expression in the gingiva. The expression levels of *MMP1* (**a**), *MMP3* (**b**), *MMP9* (**c**), *MMP13* (**d**), and *COL3A1* (**e**) were determined by quantitative real-time polymerase chain reaction. Data are presented as mean ± standard deviation of three independent experiments. * *p* < 0.05 compared with control (Ctrl).

Application of the rutin gel at the grafted bone site resulted in reduced expression of *MMP1* (12.69 ± 10.73 vs. 100 ± 20.44; Figure 2a), *MMP3* (22.94 ± 11.78 vs. 100 ± 29.23; Figure 2b), *MMP9* (40.27 ± 23.02 vs. 100 ± 19.75; Figure 2c), and *MMP13* (17.26 ± 14.13 vs. 100 ± 33.85; Figure 2d) in the gingiva of the animals as compared with the control group. Indeed, rutin treatment significantly decreased the levels of *MMP1*, *MMP3*, *MMP9*, and *MMP13* by 87%, 77%, 60%, and 83%, respectively, as compared with those in the control group. In contrast, *COL3A1* levels were significantly increased in the gingiva upon rutin treatment as compared with the non-treated control group (2230.09 ± 714.31 vs. 100 ± 20.67; Figure 2e).

### 2.2. Histomorphometry

A detailed analysis of the histological features of the bone deficit site revealed that the apical width, bone formation area, and bone marrow region were significantly increased in animals treated with rutin gel (Table 1). The remaining histological parameters analyzed showed a trend of improved outcome upon rutin treatment (*p* > 0.05). In the control and rutin treatment groups, there was no significant difference in connective regions (%). Noteworthily, the amount of connective tissue was reduced at the bone deficit site in rutin-treated animals as compared with the control group, but this difference was not statistically significant.

**Figure 3:**
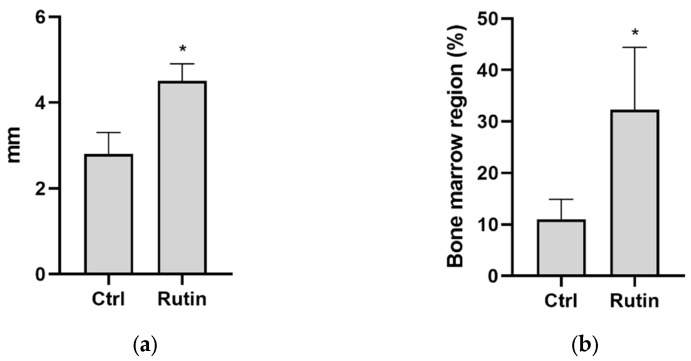
Effect of rutin gel treatment on bone formation and bone marrow at the grafted bone area. The area of newly formed bone (**a**) and percentage of bone marrow (**b**) were determined in the sliced bone sample of the grafted bone area using histological analysis techniques. Data are presented as mean ± standard deviation of three independent experiments. * *p* < 0.05 compared with control (Ctrl).

Histological analysis of sliced tissue samples of the grafted bone area was performed to determine the amount of newly formed bone (Figure 3a) and bone marrow (Figure 3b) in the two experimental groups. In line with the above-described results, newly formed bone was significantly less in the control group than in the rutin-treated group, whereas the bone marrow levels were significantly higher upon rutin treatment (Figure 3a,b).

**Figure 4:**
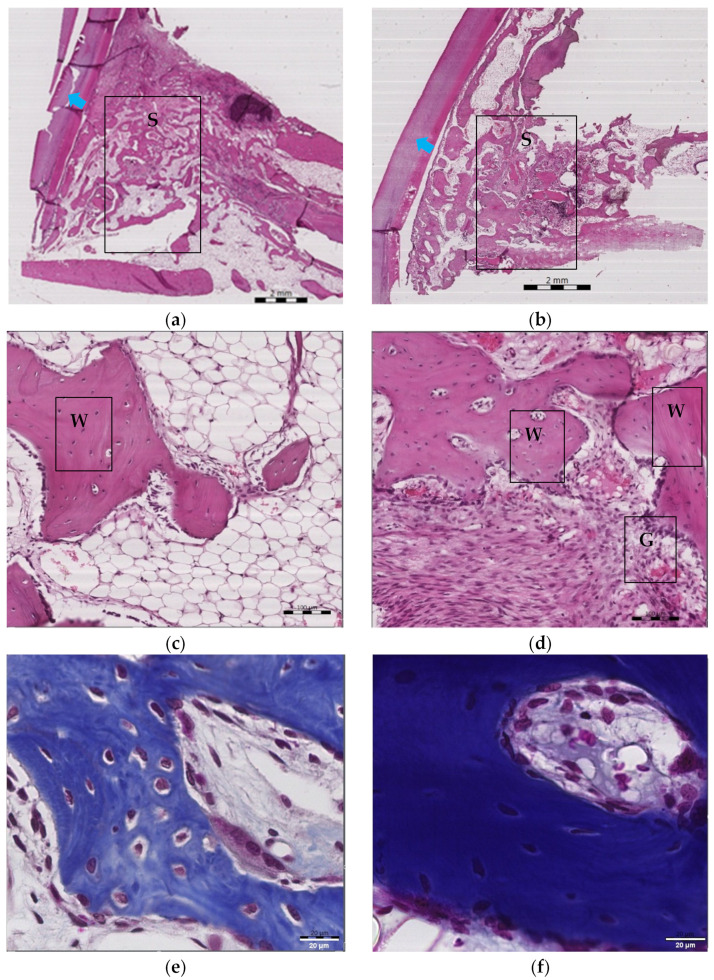
Effect of rutin gel treatment on bone formation, angiogenesis, bone marrow, and collagen fibers at the grafted bone site. Hematoxylin–eosin and Masson’s trichrome staining of jawbone samples from control (**a**,**c**,**e**) and rutin-treated (**b**,**d**,**f**) animals, respectively. In (**a**,**b**) samples, the letter S refers to the area of study where bone formation and bone marrow were measured in the lower first premolar tooth socket; additionally, the blue arrow shows the lower second premolar tooth (magnification 40×, scale bar is 2 mm). In (**c**,**d**) samples, the area of bone formation is indicated by the letter W and the area of angiogenesis is indicated by the letter G at the place of blood cell collection (magnification 100×, scale bar is 100 μm). In (**e**,**f**) samples, collagen area is indicated by blue color staining (magnification 400×, scale bar is 20 μm).

Further histological analyses showed improved bone formation and distribution and higher levels of collagen fibers as well as improved angiogenesis upon rutin treatment (Figure 4b,d,f) as compared with the control group (Figure 4a,c,e).

## 3. Discussion

This study showed that treatment with rutin can significantly reduce the levels of *MMP1*, *MMP3*, *MMP9*, and *MMP13* at bone formation sites, which agrees with previous reports. Consequently, rutin could significantly improve the desired functional outcomes [18,19,20]. A previous study reported that both MMP-1 and MMP-13 are expressed in osteoarthritic cartilage; thus, the collagenolytic activity observed in osteoarthritic cartilage is likely due to the combined activity of these two enzymes [21]. Moreover, it is believed that MMP-9 and MMP-13 play critical roles in periodontal and alveolar bone homeostasis in both health and disease conditions. In particular, MMP-9 and MMP-13 are present in inflamed areas around necrotic bones and within the epithelium [22]. According to several studies, MMP-3 is involved in the production of inflammatory cytokines and in osteoporosis and regulates bone destruction; therefore, MMP-3 may be responsible for delayed fracture healing [23].

In the present study, the rutin-treated group showed a significant increase in *COL3A1* expression, which agrees with the results of a previous study [20]. Type III collagen plays a crucial role in granulation tissue formation during tissue regeneration and repairs the damage. Additionally, it maintains the structural support required for blood vessel growth, thereby contributing to angiogenesis [24]. Moreover, type III collagen can bind to cytokines, chemokines, and growth factor receptors on the cell surface, thereby stimulating cellular activity during wound healing. Given its proprieties, type III collagen has been used to promote cell growth and differentiation during constructive remodeling. In general, this ECM component enhances the communication and interaction between cells and accelerates bone defect repair by interacting with various growth factors [9].

Histological findings showed that rutin treatment can enhance bone formation in the buccal region, which is most likely due to an interaction between the anti-inflammatory and osteogenic properties of rutin. This finding is consistent with previously published studies which showed that rutin administration to a rat model of osteoporosis in mandibular alveolar bone significantly increases bone formation [25]. Additional research also showed that rutin facilitates osteoblast differentiation and bone formation [26]. This was attributed to the upregulation of osteogenic markers and the downregulation of inflammatory cytokines [27]. Hence, rutin may be an effective treatment for bone disorders.

**Figure 5:**
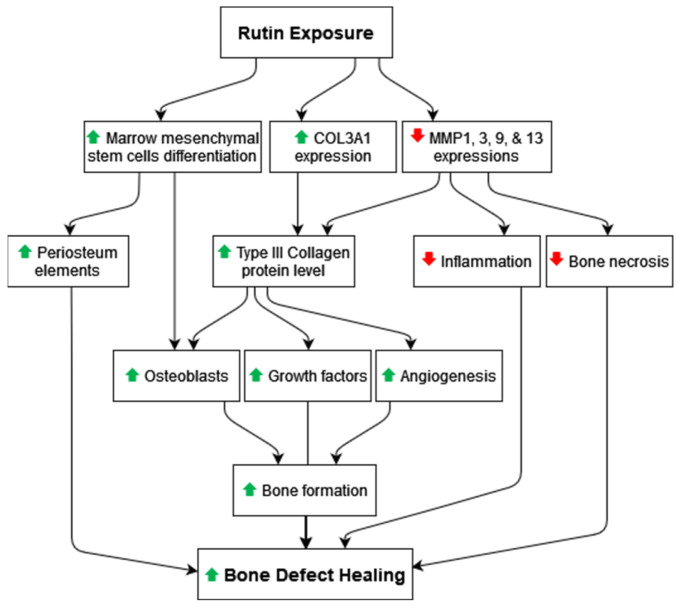
Proposed therapeutic mechanism of rutin for the acceleration and high success rates of bone grafts.

Herein, our histomorphometry results revealed a significant increase in the bone marrow area upon rutin treatment. In accordance with our findings, a previous in vivo study confirmed that rutin can promote the differentiation of marrow mesenchymal stem cells into osteoblasts [28]. During osteogenesis stages, osteoblasts deposit collagen rapidly without mineralizing it. Afterward, the mineralization rate increases to equal the collagen synthesis rate. As collagen synthesis decreases, mineralization continues until the produced collagen is fully mineralized [29]. Notably, the bone marrow was reported to play an important role in bone formation by producing osteoblasts and periosteum elements, which facilitate the connection with the surrounding tissues [30,31]. The present findings suggested that MMPs could play a role in the integration of the grafted bone with the host bone. Therefore, rutin’s potential to promote bone healing could be mediated by increasing type III collagen and decreasing MMP levels, which are known to impair tissue repair and degrade extracellular matrix components (Figure 5). Therefore, rutin can work as a suitable adjuvant agent for bone grafts to be added at the bone defect site to accelerate the grafted bone success.

Further evaluation of rutin as an adjuvant agent for bone grafts is still required to optimize the bone regeneration process. In particular, future studies should explore the most appropriate rutin dosage to use in the clinical setting and the precise molecular mechanisms underlying its therapeutic effects. The study was limited by the fact that it was conducted on healthy laboratory animals, which differed from humans in health status, diet, and environment. Therefore, clinical translation of these laboratory findings requires extensive preclinical research.

## 4. Materials and Methods

### 4.1. Preparation of the Rutin Gel

The rutin gel was prepared as previously described, but with some modifications [17]. Rutin and vehicle (without rutin) gels were prepared using the required components, which were safe and approved by the Food and Drug Administration. Briefly, ethanol (1 mL; Sigma-Aldrich, St. Louis, MO, USA) was used to solubilize the rutin powder (2.5 mg; APExBio, Houston, TX, USA), lipophilic surfactant “Span 60” (600 mg), soy lecithin (300 mg), and cholesterol (100 mg) (all from SRL, Mumbai, India). The mixture was warmed for approximately 5 min in a water bath at 70 °C with agitation. Pre-warmed distilled water (0.33 mL) was then added to the mixture and kept at the same temperature for approximately 3 min. After the solution was cooled to room temperature, the gel was stored in the dark until further use. A vehicle gel without rutin (used for the control group) was prepared by following all steps except for the addition of rutin. As part of the surgical procedure, 900 mg of bone graft was mixed with 100 mg of vehicle or rutin gel. The rutin dose used in this study was selected based on the safety margin previously reported [32,33].

### 4.2. In Vivo Experiments

In this study, six male New Zealand rabbits aged 10 months weighing between 2.75 and 3.5 kg were randomly divided into two groups: the control group received the vehicle gel, and the treated group received the rutin gel, which was combined with a demineralized freeze-dried bone allograft (DFDBA) and pericardium membrane (both from Zimmer Biomet, Palm Beach Gardens, FL, USA). A sterile standard diet and free access to water were provided to all animals under pathogen-free conditions.

### 4.3. Surgical Procedure

For general anesthesia, 40 mcg/kg dexmedetomidine HCl (used as sedative agent; APExBio) was combined with continuous isoflurane (Baxter, Deerfield, IL, USA) inhalation. Lidocaine hydrochloride (9 mg/kg; Sigma-Aldrich, St. Louis, MO, USA) was used to induce local anesthesia. The experimental defects were created using an extraoral approach through skin incisions. The first premolar to first molar teeth were incised, and a sulcular incision was made from the distal side of the lower second premolar to the mesial side of the first premolar. A vertical incision was made on the mesial side of the first premolar to achieve primary closure. Once the alveolar bone was exposed by raising the flap, lower anterior pediatric forceps were used to extract (atraumatic extraction) the mandibular first premolar PM-1 (unilaterally) from the lower teeth. Subsequently, the granulation tissue in the socket was completely cured. Randomly selected defects were assigned to the control and rutin groups. The defects were filled with 80 mg DFDBA with or without rutin gel and then covered with a pericardium collagen membrane. Interrupted sutures (resorbable polyglycolic acid, Unimed, Riyadh, Saudi Arabia) were used to close the flaps.

**Figure 6:**
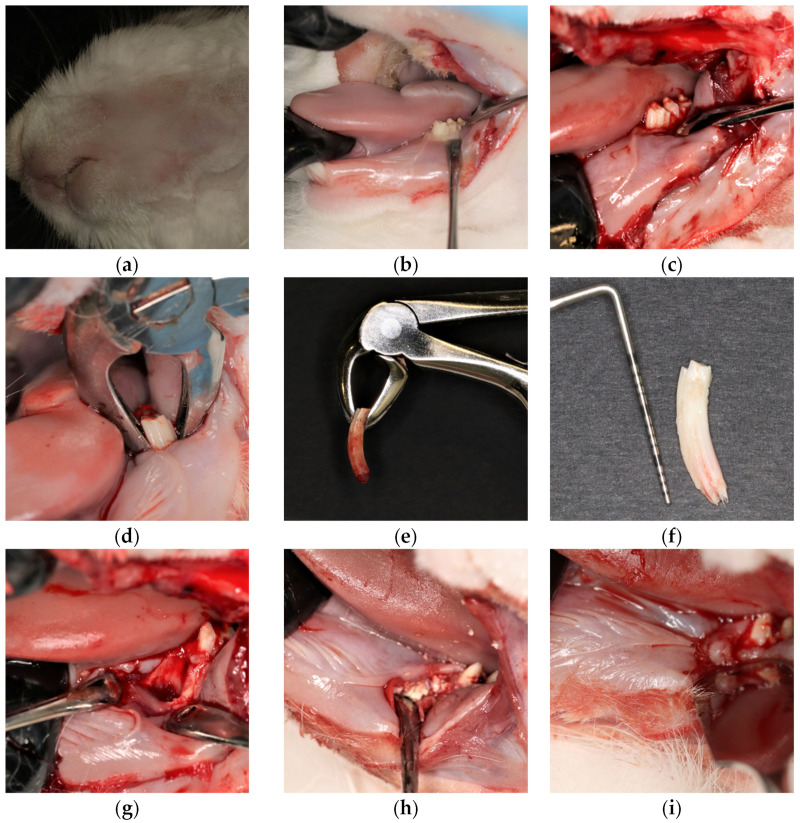

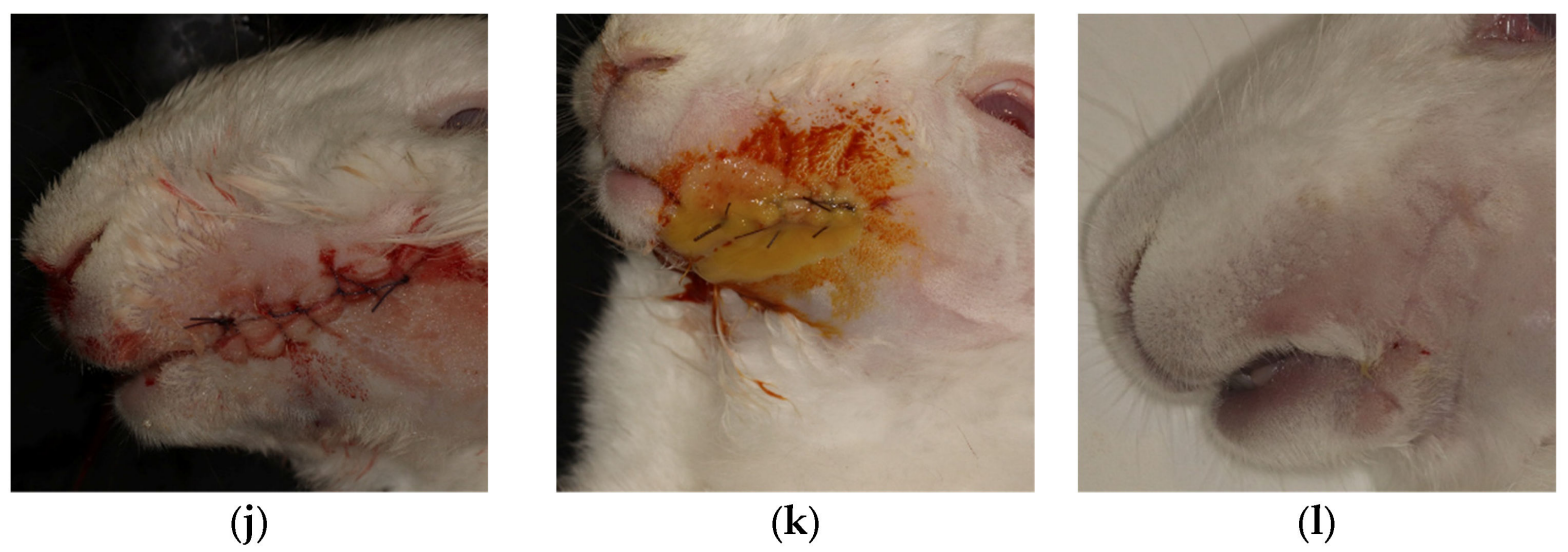
Surgical procedure. Hair was shaved after anesthesia (**a**); an external approach involves an incision in the skin exposing the surgical area (**b**); the alveolar bone was exposed around the first premolar (**c**); the extraction was performed with a lower anterior forceps (**d**); extracted lower first premolar (**e**); the lower first premolar was measured 15 mm long using the University of North Carolina-15 probe (**f**); first premolar socket (**g**); transplanted bone graft in the tooth socket and covered with a pericardium membrane (**h**); the surgical site was sutured with a resorbable type of polyglycolic acid sutures (**i**); the surgical site was sutured with extraoral sutures (**j**); an antiseptic and an antibiotic were applied to the skin after surgery (**k**); surgery site after 6 weeks (**l**).

Following surgery, all animals were provided with special food pellets. As part of their post-surgery care, the rabbits were intramuscularly administered analgesics (1 mg/kg ketoprofen; Vemedim Animal Health, Can Tho, Vietnam) and antibiotics (4 mg/kg enrofloxacin; Avico, Amman, Jordan) every 24 h for 5 days. Two weeks after surgery, the extraoral sutures were removed and the intraoral sutures were reabsorbed (Figure 6).

### 4.4. Sample Collection

The experiment was terminated after 6 weeks based on the findings of our pilot study. In this pilot study, we examined bone formation without intervention (just tooth extraction) at different time points. At the end of the sixth week, we found that the bone healing process was still incomplete, whereas the healing process and new bone formation were almost completed 12 weeks after extraction. Therefore, we chose 6 weeks as a relevant time point for comparison as we were interested in studying whether adding rutin to the bone graft accelerates bone formation or not.

**Figure 7:**
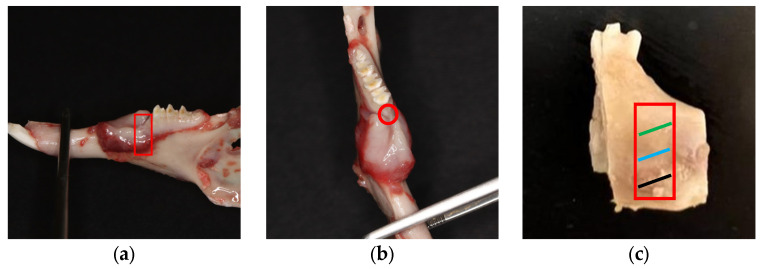
Preparation of the bone defect area for the sectioning procedure. Side view of extracted jawbone from a sacrificed rabbit with marked region of interest (**a**). Top view of extracted jawbone with marked region of interest (**b**). The area of interest was cut from the jawbone for histomorphometric analysis conducted in the marked red region. Green, blue, and black lines within the study area indicate coronal, middle, and apical widths, respectively (**c**).

The animals were euthanized with carbon dioxide after 6 weeks. Then, the jawbones were extracted from sacrificed rabbits (Figure 7a,b). Bone specimens were obtained using surgical bone saw discs from the mesial aspect of the lower first molar to the mesial aspect of the lower first premolar (Figure 7c). The bone samples were fixed in 10% formalin for examination. In addition, soft tissue specimens around the surgical site were also collected from the area adjacent to the grafted bone and stored at −80 °C. The groups were blindly assigned to independent examiners to prevent bias.

### 4.5. RNA Extraction and cDNA Synthesis

Total RNA was extracted from tissue samples using Invitrogen TRIzol reagent (Thermo Fisher Scientific, Waltham, MA, USA), according to the manufacturer’s instructions, and was quantified by measuring the absorbance at 260 nm using a Jenway Genova Nano Micro-Volume Life Science Spectrophotometer (Antylia Scientific, Vernon Hills, IL, USA). To determine the purity of the RNA, absorbance ratios of 260/280 nm (>1.8) were used. The respective cDNA was synthesized using High-Capacity cDNA Reverse Transcription Kit (Applied Biosystems, Waltham, MA, USA), in accordance with the manufacturer’s instructions. Briefly, 1.5 µg of total RNA obtained from each sample was mixed with 2 µL 10× of reverse transcriptase buffer, 0.8 µL of 25× dNTP mix, 2 µL of 10× reverse transcriptase random primers (100 mM of each), 1 µL of MultiScribe reverse transcriptase, and 4.2 µL of nuclease-free water. The final reaction mixture was maintained at 25 °C for 10 min, then heated to 37 °C for 120 min, heated again to 85 °C for 5 min, and then cooled down to 4 °C.

### 4.6. Quantification of mRNA Expression

The quantitative expression of specific mRNAs was assessed by real-time polymerase chain reaction (PCR). Briefly, 1.4 µL of cDNA was added to 25 µL PCR reaction mixture, which also contained 0.25 µL of forward and reverse primers (100 nM final concentration of each primer), 10.6 µL nuclease-free water, and 12.5 µL SYBR Green Universal Master Mix (Applied Biosystems, Waltham, MA, USA). The primers used in the study (Table 2) were synthesized by Metabion (Planegg, Germany) and based on previously published studies [34,35,36,37]. The samples were analyzed in 96-well optical reaction plates using an ABI Prism 7500 System (Applied Biosystems, Waltham, MA, USA).

As previously described [38], real-time PCR data were analyzed as relative gene expression using the 2^−∆∆Ct^ method. The target gene levels were normalized to those of *GAPDH* for comparing the fold change between the treated and untreated groups based on the following equation: fold change = 2^−∆(∆Ct)^, where ∆Ct = Ct*_target_* − Ct*_GAPDH_* and ∆(∆Ct) = ∆Ct*_treated_* − ∆Ct*_untreated_*.

### 4.7. Histomorphometry

**Figure 8:**
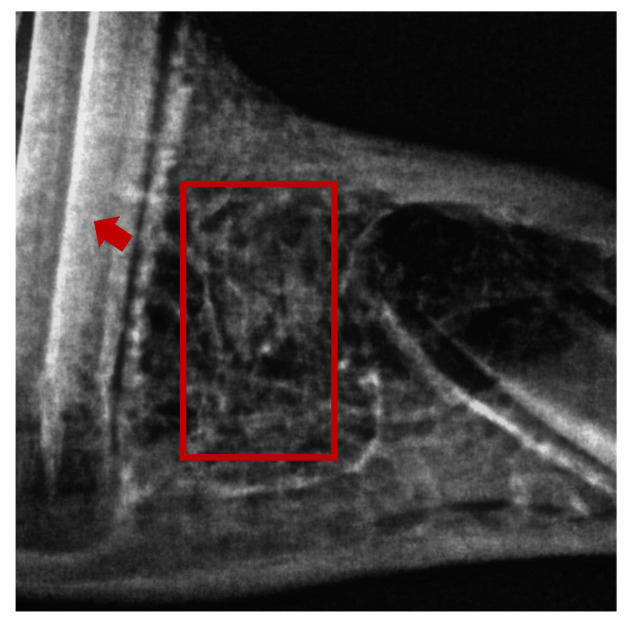
Periapical radiograph showing the most central area of the bone defect (rectangle shape) that was used for histomorphometry analysis of the lower first premolar tooth socket, in which randomly selected microscopic fields were analyzed.

The measuring of histomorphometric parameters was described, specified, and accomplished according to the work of Okada et al. (2019) [39]. Approximately 3–5 mm thick bone samples were used from each group and immediately fixed in 10% formalin. A solution of 10% formalin and 12.5% EDTA was used for 6 weeks to decalcify the samples. Each sample was then submitted within a well-labeled cassette into an automatic tissue processing machine (ASP300s; Leica Biosystems, Wetzlar, Germany). Each sample was embedded in paraffin wax, and microtomy in the sagittal direction was performed using a rotary microtome (SHUR/Cut 4500; TBS, Durham, NC, USA) [40]. Two slides were produced from each block: one for hematoxylin–eosin staining for general purposes and the other for Masson’s trichrome staining as a special staining method for connective tissue fibers [41,42]. Both stains were obtained from Nanjing Sen Beijing Jia Biological Technology Co. (Nanjing, China). To determine the most central part of the bone defect (extraction location) for histomorphometry analysis, digital periapical radiography using a hand-held X-ray generator (Genoray America, Inc., Orange, CA, USA) was performed as shown in Figure 8. Five randomly selected microscopic fields were analyzed for each sample at 400× magnification.

Each bone sample came with a tooth, and the sample was oriented such that the tooth was posterior to the study area, apical width was measured in lower position of the study area, middle width was measured in the central position of the study area, coronal width was measured in the higher position of the study area, and bone formation area was the place where bone growth was observed within the study area.

### 4.8. Statistical Analysis

The collected data were analyzed using GraphPad Prism software version 9.4.0 (GraphPad Software, Boston, MA, USA). A total of six samples divided into two groups (*n* = 3 for each group) were analyzed. Unpaired *t*-tests were used to determine significant differences between the groups. *p* < 0.05 was deemed statistically significant.

## 5. Conclusions

Rutin gel and bone grafts can be locally applied to bone defects without the need for specialized instruments. This mixture, which contains biocompatible, readily available, inexpensive, and safe substances, supports rapid bone production and promotes good bone health. Hence, rutin gel and bone grafts can be used together to treat a wide range of dental and bone-related conditions. These findings provide new foundations for the periodontics and medical fields as they describe an easy, simple, and cost-effective approach for achieving the desired results in bone grafting procedures. In future studies, bending, axial compression, and torsion tests could be used to assess bone mechanical strength following rutin treatment with a bone graft mixture.

## Figures and Tables

**Table 1:** Characterization of the histological parameters of the grafted bone site.

Histological Parameters	Control Group	Rutin-Treated Group
Coronal width (mm)	1.7 ± 0.2	1.2 ± 0.3
Middle width (mm)	0.8 ± 0.32	1.3 ± 0.05
Apical width (mm)	4.5 ± 0.4	6.3 ± 0.4 *
Bone formation area diameter (mm)	2.8 ± 0.5	4.5 ± 0.4 *
Mineralized bone region (%)	26.6 ± 6.4	34.2 ± 4.5
Bone marrow region (%)	11 ± 3.9	32.3 ± 12.1 *
Connective tissue region (%)	46.9 ± 10.9	33.0 ± 8.7

Data are presented as mean ± standard deviation (*n* = 3 per group). * *p* < 0.05 compared with the control group as determined by Student’s *t*-test.

**Table 2:** Sequences of the primers used for real-time polymerase chain reaction analysis.

Gene	Forward Primer (5′–3′)	Reverse Primer (5′–3′)
*MMP1*	CCTGATGTGGCTCAGTTCGT	GTCCACATCTGCCCTTGACA
*MMP3*	TGGACCTGGAAATGTTTTGG	ATCAAAGTGGGCATCTCCAT
*MMP9*	ACGGCCGACTATGACACC	TTGCCGTCCTGGGTGTAG
*MMP13*	CCTCTTCTTCTCCGGAAACC	GGTAGTCTTGGTCCATGGTATGA
*COL3A1*	GCAGGGACTCCAGGTCTTAGAGG	CGTGTTCACCTCTCTCTCCCAGGG
*GAPDH*	CACAGTTTCCATCCCAGACC	TGGTTTCATGACAAGGTAGGG

## Data Availability

All the data are given in the manuscript.

## References

[1] Titsinides S., Agrogiannis G., Karatzas T. (2019). Bone grafting materials in dentoalveolar reconstruction: A comprehensive review. Jpn. Dent. Sci. Rev..

[2] Bouxsein M.L., Turek T.J., Blake C.A., D’Augusta D., Li X., Stevens M., Seeherman H.J., Wozney J.M. (2001). Recombinant Human Bone Morphogenetic Protein-2 Accelerates Healing in a Rabbit Ulnar Osteotomy Model. J. Bone Joint Surg. Am..

[3] El Bialy I., Jiskoot W., Reza Nejadnik M. (2017). Formulation, Delivery and Stability of Bone Morphogenetic Proteins for Effective Bone Regeneration. Pharm. Res..

[4] Shimono K., Oshima M., Arakawa H., Kimura A., Nawachi K., Kuboki T. (2010). The effect of growth factors for bone augmentation to enable dental implant placement: A systematic review. Jpn. Dent. Sci. Rev..

[5] Einhorn T.A., Gerstenfeld L.C. (2015). Fracture healing: Mechanisms and interventions. Nat. Rev. Rheumatol..

[6] Kusano K., Miyaura C., Inada M., Tamura T., Ito A., Nagase H., Kamoi K., Suda T. (1998). Regulation of Matrix Metalloproteinases (MMP-2, -3, -9, and -13) by Interleukin-1 and Interleukin-6 in Mouse Calvaria: Association of MMP Induction with Bone Resorption. Endocrinology.

[7] Bonnans C., Chou J., Werb Z. (2014). Remodelling the extracellular matrix in development and disease. Nat. Rev. Mol. Cell Biol..

[8] Elgezawi M., Haridy R., Almas K., Abdalla M.A., Omar O., Abuohashish H., Elembaby A., Christine Wölfle U., Siddiqui Y., Kaisarly D. (2022). Matrix Metalloproteinases in Dental and Periodontal Tissues and Their Current Inhibitors: Developmental, Degradational and Pathological Aspects. Int. J. Mol. Sci..

[9] Cheng P., Li D., Gao Y., Cao T., Jiang H., Wang J., Li J., Zhang S., Song Y., Liu B. (2018). Prevascularization promotes endogenous cell-mediated angiogenesis by upregulating the expression of fibrinogen and connective tissue growth factor in tissue-engineered bone grafts. Stem Cell. Res. Ther..

[10] Li S., Li Y., Jiang Z., Hu C., Gao Y., Zhou Q. (2022). Efficacy of total flavonoids of Rhizoma drynariae on the blood vessels and the bone graft in the induced membrane. Phytomedicine.

[11] Benjamin M.M., Khalil R.A. (2012). Matrix metalloproteinase inhibitors as investigative tools in the pathogenesis and management of vascular disease. Exp. Suppl..

[12] Kumar G.B., Nair B.G., Perry J.J.P., Martin D.B.C. (2019). Recent insights into natural product inhibitors of matrix metalloproteinases. Medchemcomm.

[13] Ganeshpurkar A., Saluja A.K. (2017). The Pharmacological Potential of Rutin. Saudi Pharm. J..

[14] Sharma S., Ali A., Ali J., Sahni J.K., Baboota S. (2013). Rutin: Therapeutic potential and recent advances in drug delivery. Expert Opin. Investig. Drugs.

[15] Younis T., Jabeen F., Hussain A., Rasool B., Raza Ishaq A., Nawaz A., El-Nashar H.A.S., El-Shazly M. (2023). Antioxidant and Pulmonary Protective Potential of Fraxinus xanthoxyloides Bark Extract against CCl4-Induced Toxicity in Rats. Chem. Biodivers..

[16] Abdelghffar E.A.R., Mostafa N.M., El-Nashar H.A.S., Eldahshan O.A., Singab A.N.B. (2022). Chilean pepper (*Schinus polygamus*) ameliorates the adverse effects of hyperglycaemia/dyslipidaemia in high fat diet/streptozotocin-induced type 2 diabetic rat model. Ind. Crops Prod..

[17] Pinzaru I., Tanase A., Enatescu V., Coricovac D., Bociort F., Marcovici I., Watz C., Vlaia L., Soica C., Dehelean C. (2021). Proniosomal Gel for Topical Delivery of Rutin: Preparation, Physicochemical Characterization and In Vitro Toxicological Profile Using 3D Reconstructed Human Epidermis Tissue and 2D Cells. Antioxidants.

[18] Liu L.L., Zhang Y., Zhang X.F., Li F.H. (2018). Influence of rutin on the effects of neonatal cigarette smoke exposure-induced exacerbated MMP-9 expression, Th17 cytokines and NF-κB/iNOS-mediated inflammatory responses in asthmatic mice model. Korean J. Physiol. Pharmacol..

[19] Chen X., Yu M., Xu W., Zou L., Ye J., Liu Y., Xiao Y., Luo J. (2021). Rutin inhibited the advanced glycation end products-stimulated inflammatory response and extra-cellular matrix degeneration via targeting TRAF-6 and BCL-2 proteins in mouse model of osteoarthritis. Aging.

[20] Her Y., Lee T.K., Kim J.D., Kim B., Sim H., Lee J.C., Ahn J.H., Park J.H., Lee J.W., Hong J. (2020). Topical Application of Aronia melanocarpa Extract Rich in Chlorogenic Acid and Rutin Reduces UVB-Induced Skin Damage via Attenuating Collagen Disruption in Mice. Molecules.

[21] Mitchell P.G., Magna H.A., Reeves L.M., Lopresti-Morrow L.L., Yocum S.A., Rosner P.J., Geoghegan K.F., Hambor J.E. (1996). Cloning, expression, and type II collagenolytic activity of matrix metalloproteinase-13 from human osteoarthritic cartilage. J. Clin. Investig..

[22] Soundia A., Hadaya D., Esfandi N., Gkouveris I., Christensen R., Dry S.M., Bezouglaia O., Pirih F., Nikitakis N., Aghaloo T. (2018). Zoledronate Impairs Socket Healing after Extraction of Teeth with Experimental Periodontitis. J. Dent. Res..

[23] Hu Y., Zhang T., Huang H., Cheng W., Lai Y., Bai X., Chen J., Yue Y., Zheng Z., Guo C. (2018). Fracture healing in a collagen-induced arthritis rat model: Radiology and histology evidence. J. Orthop. Res..

[24] Garnero P. (2015). The Role of Collagen Organization on the Properties of Bone. Calcif. Tissue Int..

[25] Abdul-Fattah Baraka N., Fathallah Ahmed N., Ismail Hussein S. (2022). The effect of Rutin hydrate on Glucocorticoids induced osteoporosis in mandibular alveolar bone in Albino rats (Radiological, histological and histochemical study). Saudi Dent. J..

[26] Lee H.-H., Jang J.-W., Lee J.-K., Park C.-K. (2020). Rutin Improves Bone Histomorphometric Values by Reduction of Osteoclastic Activity in Osteoporosis Mouse Model Induced by Bilateral Ovariectomy. J. Korean Neurosurg. Soc..

[27] Chen X., Hu C., Wang G., Li L., Kong X., Ding Y., Jin Y. (2013). Nuclear factor-κB modulates osteogenesis of periodontal ligament stem cells through competition with β-catenin signaling in inflammatory microenvironments. Cell Death Dis..

[28] Xiao Y., Wei R., Yuan Z., Lan X., Kuang J., Hu D., Song Y., Luo J. (2019). Rutin suppresses FNDC1 expression in bone marrow mesenchymal stem cells to inhibit postmenopausal osteoporosis. Am. J. Transl. Res..

[29] Moreira C.A., Dempster D.W., Baron R., Feingold K.R., Anawalt B., Blackman M.R., Boyce A., Chrousos G., Corpas E., de Herder W.W., Dhatariya K., Dungan K., Hofland J. (2000). Anatomy and ultrastructure of bone–histogenesis, growth and remodeling. Endotext.

[30] Colnot C. (2009). Skeletal cell fate decisions within periosteum and bone marrow during bone regeneration. J. Bone Miner. Res..

[31] Petrescu H.P., Dinu G., Nodiţi G., Berceanu-Văduva M., Bratu D.C., Vermeşan D. (2014). Experimental morphologic and radiologic study of the integration of bone grafts into the host tissue and of the dynamics of the graft-receptor interface. Rom. J. Morphol. Embryol..

[32] da Silva J., Herrmann S.M., Heuser V., Peres W., Possa Marroni N., González-Gallego J., Erdtmann B. (2002). Evaluation of the genotoxic effect of rutin and quercetin by comet assay and micronucleus test. Food Chem. Toxicol..

[33] Cristina Marcarini J., Ferreira Tsuboy M.S., Cabral Luiz R., Regina Ribeiro L., Beatriz Hoffmann-Campo C., Ségio Mantovani M. (2011). Investigation of cytotoxic, apoptosis-inducing, genotoxic and protective effects of the flavonoid rutin in HTC hepatic cells. Exp. Toxicol. Pathol..

[34] Saulnier N., Viguier E., Perrier-Groult E., Chenu C., Pillet E., Roger T., Maddens S., Boulocher C. (2015). Intra-articular administration of xenogeneic neonatal Mesenchymal Stromal Cells early after meniscal injury down-regulates metalloproteinase gene expression in synovium and prevents cartilage degradation in a rabbit model of osteoarthritis. Osteoarthr. Cartil..

[35] Inoue H., Arai Y., Kishida T., Terauchi R., Honjo K., Nakagawa S., Tsuchida S., Matsuki T., Ueshima K., Fujiwara H. (2015). Hydrostatic pressure influences HIF-2 alpha expression in chondrocytes. Int. J. Mol. Sci..

[36] Ishibashi H., Tonomura H., Ikeda T., Nagae M., Sakata M., Fujiwara H., Tanida T., Mastuda K.-I., Kawata M., Kubo T. (2016). Hepatocyte growth factor/c-met promotes proliferation, suppresses apoptosis, and improves matrix metabolism in rabbit nucleus pulposus cells in vitro. J. Orthop. Res..

[37] González J.C., López C., Álvarez M.E., Pérez J.E., Carmona J.U. (2016). Autologous leukocyte-reduced platelet-rich plasma therapy for Achilles tendinopathy induced by collagenase in a rabbit model. Sci. Rep..

[38] Livak K.J., Schmittgen T.D. (2001). Analysis of relative gene expression data using real-time quantitative PCR and the 2−ΔΔCT method. Methods.

[39] Okada M., Matsuura T., Akizuki T., Hoshi S., Shujaa Addin A., Fukuba S., Izumi Y. (2019). Ridge preservation of extraction sockets with buccal bone deficiency using poly lactide-co-glycolide coated β-tricalcium phosphate bone grafts: An experimental study in dogs. J. Periodontol..

[40] Hamad A.M., Ahmed H.G. (2018). Association of some carbohydrates with estrogen expression in breast lesions among Sudanese females. J. Histotechnol..

[41] Hamad A., Ahmed H. (2016). Association of connective tissue fibers with estrogen expression in breast lesions among Sudanese females. Int. Clin. Pathol. J.

[42] Suvarna S.K., Layton C., Bancroft J.D. (2019). Theory and Practice of Histological Techniqueseighth.

